# Periodic discharges in veterinary electroencephalography—A visual review

**DOI:** 10.3389/fvets.2023.1037404

**Published:** 2023-01-26

**Authors:** Marguerite F. Knipe, William W. Bush, Kristen E. Thomas, D. Colette Williams

**Affiliations:** ^1^Department of Surgical and Radiological Sciences, School of Veterinary Medicine, University of California, Davis, Davis, CA, United States; ^2^Bush Veterinary Neurology Service, Leesburg, VA, United States; ^3^School of Veterinary Medicine, William R. Pritchard Veterinary Medical Teaching Hospital, University of California, Davis, Davis, CA, United States

**Keywords:** EEG, canine, feline, encephalopathy, epilepsy, seizures, status epilepticus

## Abstract

First described in human EEG over 60 years ago, there are very few examples of periodic discharges in the veterinary literature. They are associated with a wide variety of etiologies, both intracranial and systemic, making interpretation challenging. Whether these patterns are indicative of ictal, interictal, or postictal activity is a matter of debate and may vary depending on the clinical features in an individual patient. Periodic discharges have a repeated waveform occurring at nearly regular intervals, with varying morphology of individual discharges from simple sharp waves or slow waves to more complex events. Amplitudes, frequencies, and morphologies of the discharges can fluctuate, occasionally evolving, or resolving over time. This study presents a visual review of several veterinary cases with periodic discharges on EEG similar to those described in human EEG, and discusses the current known pathophysiology of these discharges.

## 1. Introduction

In human medicine, electroencephalography (EEG), and particularly continuous EEG (cEEG) has become an essential part of monitoring brain function in critically ill patients, both with and without known brain disease (1, 2). First described in human EEG over 60 years ago (3, 4), periodic discharges are noted with varying frequency depending on the patient population, however, they are being documented with increasing frequency in the continuous critical care EEG recordings (2, 5–7).

The first reported periodic pattern was termed PLEDs (periodic lateralized epileptiform discharges), and these discharges were often noted in association with seizures and acute structural brain disease in humans, particularly stroke (3, 8–11). In 2005, the American Clinical Neurophysiology Society (ACNS) recommended removing the word “epileptiform” from the terminology for periodic discharges to be more strictly descriptive of the waveforms, and to avoid clinical connotations or to suggest etiology (12). Subsequent terminology guidelines upheld and elaborated on standardized descriptions for periodic discharges to facilitate consistency (13–15) (Box 1). This alteration in terminology reflects the research over the years supporting that periodic discharges (PDs) are not always related to seizures (i.e. not “epileptiform”), but are sequelae to a focally or globally injured or otherwise dysfunctional cerebrum (10, 11, 16, 17) from a wide variety of causes, including seizures/status epilepticus (6), systemic metabolic disease (1, 18), hypothermia (19), and administration of (20, 21) or withdrawal from (7) anesthetic drugs.

Box 1 **Definitions (14, 15).**

*Generalized (G)*: any bilaterally synchronous and symmetric pattern, even if it has a restricted field (e.g., bifrontal).

*Lateralized (L)*: unilateral OR bilateral but clearly and consistently higher amplitude in one hemisphere (bilateral asymmetric); OR bilateral but with a consistent lead-in from the same side (bilateral asynchronous).

*Bilateral Independent (BI)*: two independent (and therefore asynchronous) lateralized patterns with one in each hemisphere, with both patterns occurring simultaneously, i.e., two independent patterns occurring at the same time (overlapping in time) rather than sequentially (one starting after the other stops).

*Unilateral Independent (UI)*: two independent (and therefore asynchronous) periodic or rhythmic patterns in the same hemisphere, with both patterns occurring simultaneously i.e., two independent patterns occurring at the same time (overlapping in time) rather than sequentially (one starting after the other stops).

*Multifocal (Mf)*: at least three independent lateralized patterns, with at least one in each hemisphere, with all three or more patterns occurring simultaneously.

*Periodic:* Repetition of a waveform with relatively uniform morphology and duration with a clearly discernible interdischarge-interval between consecutive waveforms and recurrence of the waveform at nearly regular intervals.

*Discharges*: Waveforms lasting < 0.5 s, regardless of number of phases, or waveforms ≥0.5 s with no more than 3 phases. This is as opposed to Bursts, defined as waveforms lasting ≥0.5 s and having at least 4 phases. Discharges and bursts must clearly stand out from the background activity

“*Plus” modifiers*: additional features causing the PD pattern to be more ictal-appearing than the same PD pattern would without the modifier

- +*F*: superimposed Fast activity as part of the PD pattern and not just background activity
- +*R*: *R*hythmic or quasi-rhythmic delta (< 4 Hz) activity superimposed over the PD pattern
- +*FR*: both modifiers are present in the PD pattern

*Fluctuating*: ≥3 changes in frequency, each < 1 min length

*Evolving*: ≥2 changes in frequency in the same direction, e.g., 1 → 1.5 → 2 Hz

The concept of the “ictal-interictal continuum” (IIC) recognizes that many patients with status epilepticus or acute brain injury from any cause may alternate between ictal and interictal—but encephalopathic—states, and it is within the IIC where many of the rhythmic and periodic patterns lie (2, 10, 22–24).

Veterinary medicine has few reports on EEG beyond describing seizures and ictal patterns (25, 26). In spite of EEG abnormalities determining the highest tier of confidence for diagnosis of idiopathic epilepsy in dogs (27), its use is currently uncommon beyond emergency referral or university practices (28–31). With increased access to digital EEG, its use in monitoring and managing veterinary patients with status epilepticus (SE) or non-convulsive status epilepticus (NCSE) is also increasing (28–30).

This descriptive paper presents visual examples of PDs in veterinary EEG paralleling those described in human EEG, and reviews the human literature on what is known and still unknown about PDs. The goal of this report is to increase recognition and understanding of these periodic patterns on veterinary EEG, especially for monitoring in the critical setting.

## 2. Case selection

Canine and feline EEG cases from two veterinary facilities (UC Davis, Bush Veterinary Neurology Services—multiple locations) from 2003 to 2021 were retrospectively identified by authors (DCW, WWB, MFK) for presence of periodic discharges on the recording as defined in Box 1. Subdermal needle electrodes were used in all cases, and recording montages and electrode placement for each facility were slightly different (Supplementary Figures 1, 2), with concurrent electrocardiogram (ECG). Recordings were reviewed primarily in each institution's bipolar montage (Supplementary Figures 1, 2) (29, 32); remontaging to view a referential montage was also done to aid localization of events (33). The referential montage utilized ‘linked ears' (A1 & A2; Supplementary Figure 1) as the second input.

Cases were chosen exclusively on the visual identification of PDs occurring for 25% or more of the EEG recording. All cases with PDs were clinical patients, for which the EEG was done at the request of the primary clinician for one of three reasons: documenting ictal or interictal abnormalities in a patient with known seizures, evaluating for possible non-convulsive status epilepticus (NCSE), or assessment of cortical function in a patient with stupor or coma. Specific sedation drugs to obtain the EEG were used as clinically needed to assist in electrode placement and dampen muscle artifact. This ranged from no medications (obtunded/stuporous patients), to only whatever antiseizure medications (ASMs) the patient was receiving for management, or specific intravenous (IV) administration of various sedative drugs to obtain a diagnostic EEG recording. Characteristics of the PDs noted were waveform morphology, frequency of the discharges, fluctuation or evolution of either morphology or frequency, and the localization of the discharge, either generalized (GPD) or lateralized (LPD) (Box 1).

## 3. Results

### 3.1. Localization/polarity

**Figure 1:**
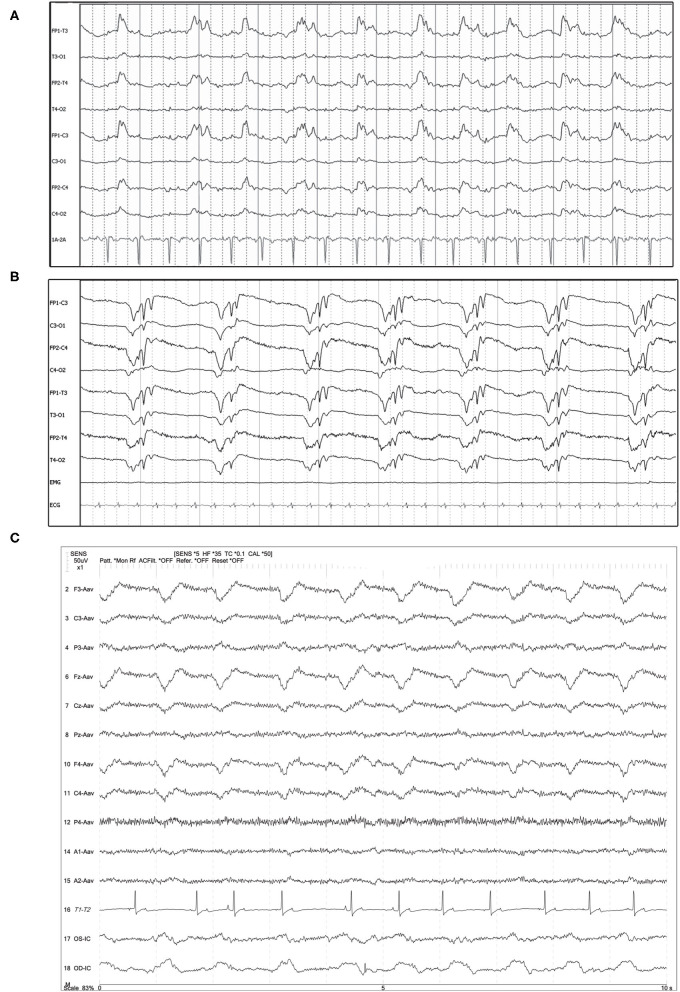
Three examples of generalized periodic discharges (GPDs). PDs are noted predominantly frontal and in both hemispheres in each case. **(A)** Ten year old mixed breed dog with several year history of seizures and recent cluster seizures. Polyphasic GPDs, synchronous and bilateral, with slightly higher amplitude in the left hemisphere (Bipolar montage; HF 70 Hz, sensitivity 5 uV/mm). **(B)** Four year old domestic longhair cat with otogenic bacterial encephalitis and seizures. Polyphasic GPDs (Bipolar montage; HF 70 Hz, sensitivity 7 uV/mm). **(C)** Thirteen year old Bichon frise with MUO and recent cluster seizures (Referential montage; HF 35 Hz, sensitivity 5 uV/mm). The amplitude of the discharge is slightly lower in F4 than F3 & Fz, although the timing of the PD is synchronous in the frontal leads. MUO, meningoencephalitis of unknown origin.

**Figure 2:**
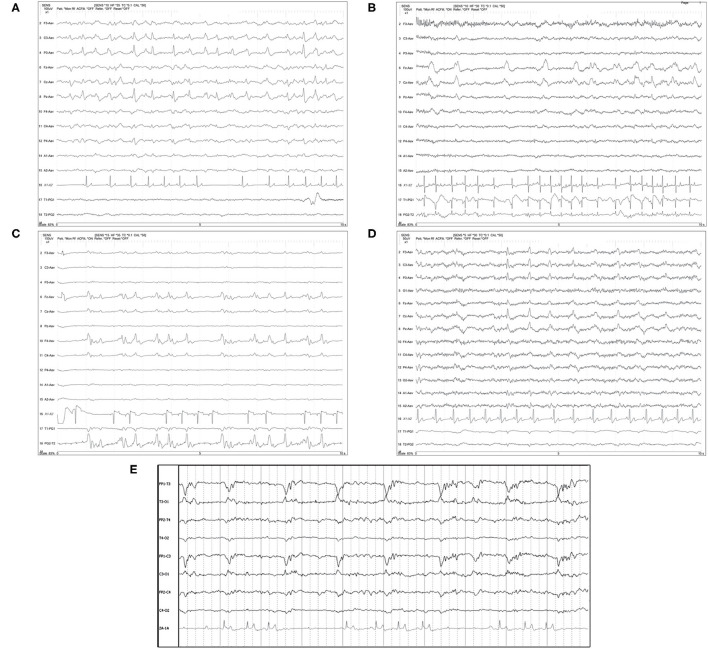
Referential **(A–D)** and bipolar **(E)** montages of five cases with lateralized periodic discharges (LPDs). **(A)** Left parietal LPDs in a 12 year old pug with cluster seizures, normal brain MRI (HF 35 Hz, sensitivity 7 uV/mm). **(B)** Frontal midline LPDs in a 14 year old Chihuahua mix with cluster seizures secondary to hypoglycemia and an insulin-secreting tumor (HF 30 Hz, sensitivity 10 uV/mm). **(C)** Right frontal LPDs in an 11 year old miniature Pinscher with recent SE and history of transfrontal craniotomy 6 months previously for right olfactory/frontal lobe meningioma (HF 35 Hz, sensitivity 15 uV/mm). **(D)** Left hemisphere LPDs in an 11 year old Labrador with recent cluster seizures and history of craniotomy 6 months previously for left occipital meningioma (HF 30 Hz, sensitivity 5 uV/mm). **(E)** Left hemisphere polyphasic LPDs (phase reversal at T3 and C3) in an 8 year old soft-coated Wheaton terrier with metastatic neoplasia (HF 35 Hz, sensitivity 5 uV/mm).

The prominent feature of visual identification of PDs is the repeated waveform of relatively uniform morphology, prominently standing out from the background, occurring at a regular or nearly regular interval, with a discernible inter-discharge interval present between consecutive waveforms (Box 1). Localization of the PDs was noted as either generalized (GPD) if the discharge was synchronous and symmetric in the hemispheres (Figure 1), or lateralized (LPD) if the discharge localized to one hemisphere or lobe, either exclusively or primarily (Figure 2). GPDs may have a frontal predominance (14), if both right and left sides are similarly affected (Figure 1). LPDs were localized on bipolar montage with instrumental phase reversal, and/or on referential montage by assessing the channels with the highest amplitude (Figure 2). Only GPD or LPD patterns were recognized in our cases: no examples of other described PDs in human medicine were noted [Bilateral Independent (BIPDs), Unilateral Independent (UIPDs), or Multifocal (MfPDs)] (Box 1).

### 3.2. Waveform morphology

**Figure 3:**
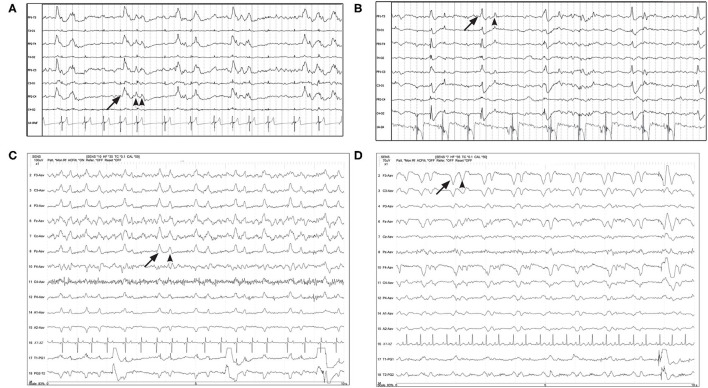
Four examples of periodic discharges, where the morphology of the PD (solid arrow) included a second or third waveform (arrowheads), with a consistent temporal relationship to the initial discharge, like a doublet or triplet. **(A)** Eleven year old Chihuahua with SE (Bipolar montage; HF 70 Hz, sensitivity 20 uV/mm). **(B)** Two year old bichon mix with progressive partial seizures and MUO (Bipolar montage; HF 70 Hz, sensitivity 10 uV/mm). **(C)** Three year old terrier mix with cluster seizures, normal brain MRI, likely idiopathic epilepsy (Referential montage; HF 35 Hz, sensitivity 10 uV/mm). **(D)** Five year old Basset hound presented with heat stroke, stuporous, multiple organ dysfunction, no seizures documented (Referential montage; HF 35 Hz, sensitivity 7 uV/mm).

**Figure 4:**
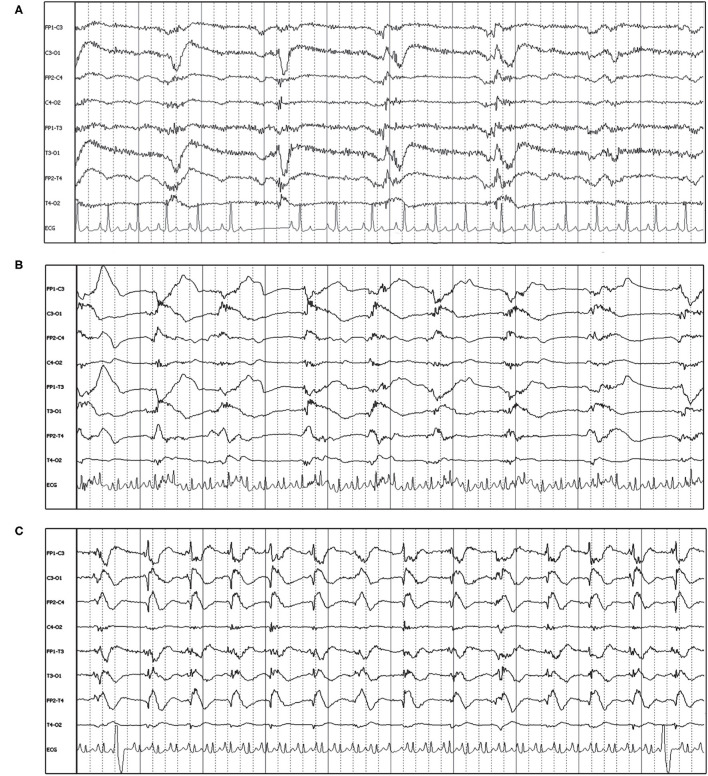
EEG epochs over the course of 45 min in a 10 year old Boxer with hypoglycemia and SE demonstrating evolution in PD frequency as well as change in morphology to a more ictal-appearing pattern. Bipolar montage. **(A)** PD frequency is about 0.5 Hz, some with a triphasic morphology. There is no apparent phase reversal, but the discharge has the highest amplitude on the left side, and end-of-chain would localize to O1 (HF 35 Hz, sensitivity 7 uV/mm). **(B)** Frequency increases to almost 1 Hz, along with a change in morphology and increased amplitude—note the change in sensitivity. Discharges are still lateralized (LPDs), with phase reversal noted in the left hemisphere at T3 and C3 (HF 15 Hz, sensitivity 50 uV/mm). **(C)** Frequency of PDs continues to increase to 1.5 Hz, becoming more generalized and developing a spike-wave morphology, and the clinical diagnosis of electrographic seizure (ES) was made (HF 15 Hz, sensitivity 30 uV/mm).

**Figure 5:**
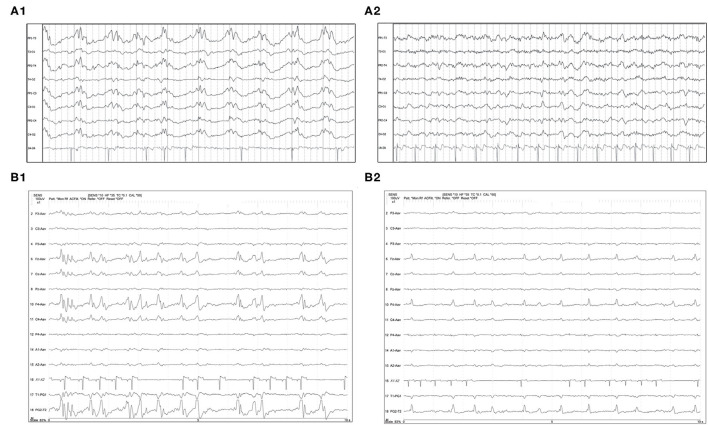
Two examples of resolution **(A1, A2)** or apparent improvement **(B1, B2)** of PDs on serial EEGs. **(A1)** Three year old Labrador mix with cluster seizures and MUO. Polyphasic GPDs frequency about 1Hz (bipolar montage; HF 70 Hz, sensitivity 3 uV/mm). **(A2)** Recheck EEG of the same dog **(A1)** on the following day showing resolution of the PDs and apparently normal EEG (bipolar montage; HF 15 Hz, sensitivity 2 uV/mm). **(B1)** Eleven year old Miniature Pinscher with recent SE (same dog as Figure 2C). Polyphasic right frontal LPDs (referential montage; HF 35 Hz, sensitivity 10 uV/mm). **(B2)** Recheck EEG of the same dog **(B1)** on the following day. The right frontal LPDs are still present, but are no longer polyphasic, and have a lower amplitude than noted in **(B1)** (referential montage; HF 35 Hz, sensitivity 10 uV/mm). MUO, meningoencephalitis of unknown origin.

**Figure 6:**
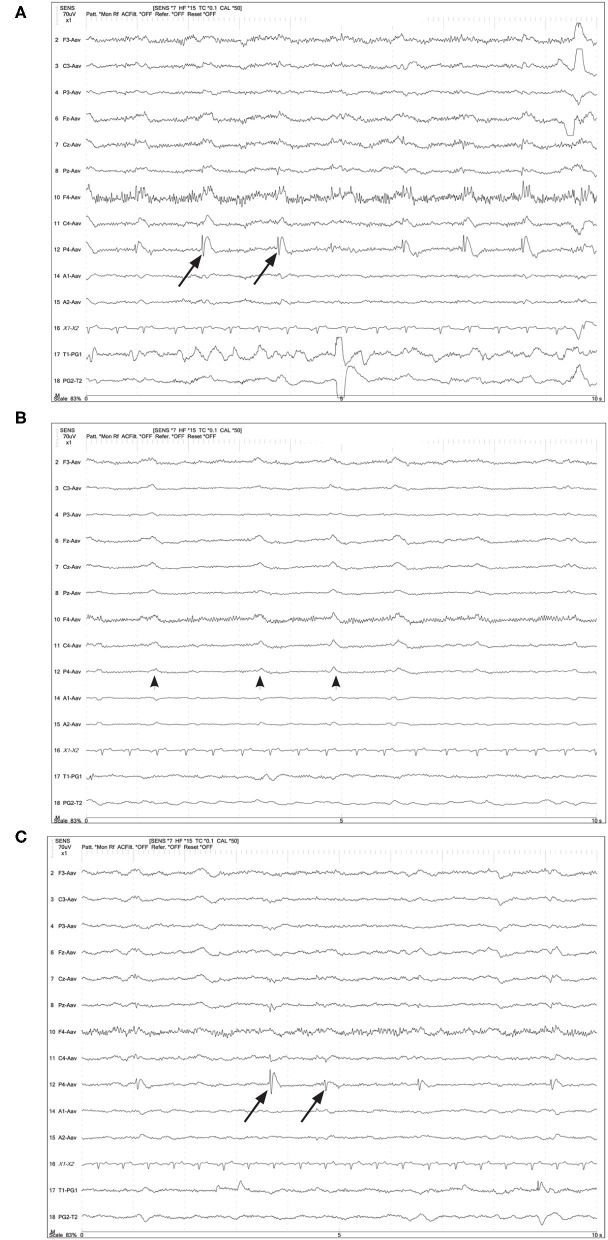
Four year old German Shepherd with cluster seizures and HE. Referential montage; HF 15 Hz, sensitivity 5 uV/mm in all panels. **(A)** Right parietal LPDs (arrows) with spike-wave morphology, immediately prior to IV midazolam (MDZ). **(B)** Shortly after IV MDZ, the LPD morphology is changed to more blunted and lower amplitude (arrowheads), but still noted on the right hemisphere and midline chain. **(C)** Ten minutes post-MDZ, the LPDs at P4 (arrows) have returned to the spike-wave morphology.

Periodic discharge morphology was typically a solitary, static waveform by definition (Figures 1, 2), and there were also instances of paired waveforms, like a doublet or triplet, that had consistent temporal associations with each other (Figure 3). In some cases, morphology of the PD changed: either becoming more polyphasic, or less polyphasic or “simpler.” Changes in PD morphology might be noted within the same recording (Figure 4), or on subsequent recheck EEGs (Figure 5), and could change spontaneously (Figure 4), or in response to an intervention, like ASM administration (Figure 6).

### 3.3. Waveform modifiers

**Figure 7:**
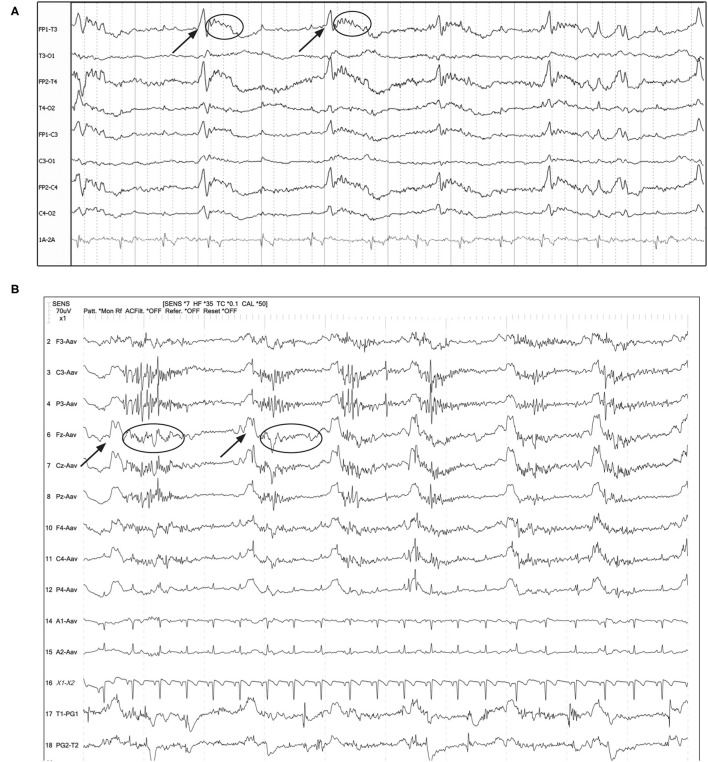
Examples of GPDs (arrow) with apparent superimposed fast activity (circle), similar to the “+F” modifier (Box 1). **(A)** Seven year old Swiss Mountain Dog presenting in SE, post-ictal changes noted on MRI, likely idiopathic epilepsy (Bipolar montage; HF 70 Hz, sensitivity 2 uV/mm). **(B)** Two year old French bulldog with cluster seizures and MUO, 10 min post-MDZ IV bolus. A left ear twitch was noted AFTER each PD and fast activity, in the interdischarge interval. ECG artifact is noted in channels 14 & 15 (Referential montage; HF 35 Hz, sensitivity 7 uV/mm). MUO, meningoencephalitis of unknown origin.

Additional characteristics of PD waveforms, when present in humans, increase the suspicion for association with or development of seizures (Box 1). Fast activity or rhythmic activity (designated “+F” and “+R,” respectively), superimposed on the PDs are correlated with an increased likelihood of seizures in humans (15). In some of our cases, there were PDs with superimposed fast activity that could be considered to have +F features (Figure 7). In the two EEGs depicted in Figure 7, both dogs had recordings done in close temporal proximity to their seizures.

### 3.4. Waveforms with triphasic morphology

**Figure 8:**
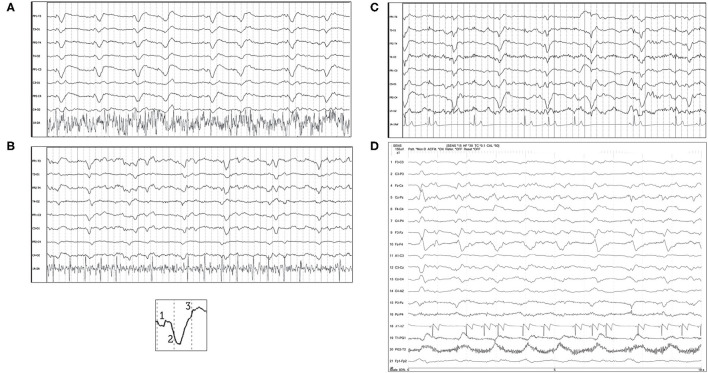
Bipolar montages of four cases with GPDs with triphasic morphology. The initial phase in each case is very small, with prominent second and third phases (inset figure). **(A)** Three year old Australian Shepherd mix with seizures and cerebral intra-axial mass on MRI (HF 70 Hz, sensitivity 7 uV/mm). **(B)** Five year old American Staffordshire terrier with cluster seizures, polycythemia (HF 15 Hz, sensitivity 3 uV/mm). **(C)** Five year old Scottish terrier with cluster seizures, likely idiopathic epilepsy (HF 30 Hz, sensitivity 15 uV/mm). **(D)** Four year old wirehair terrier with seizures and HE (HF 30 Hz, sensitivity 15 uV/mm).

“Triphasic waves” in human EEG are noted as a characteristic GPD with a small upward phase, a large, broad, downward phase, followed by a third larger upward phase (Figure 8 inset), with a relatively low frequency (0.5–2 Hz). Current human nomenclature describes these waveforms as “GPDs with triphasic morphology” (15). Examples of this type of waveform morphology are seen in Figure 8, with underlying etiologies including both metabolic encephalopathy as well as idiopathic epilepsy.

### 3.5. Frequency

The frequency of the PDs was primarily static for the individual recording (Figures 1–3, 5, 7, 8), and in some instances was fluctuating (Box 1) between higher and lower frequencies (Figure 9), or evolving (Box 1), and evolving PDs were noted to progress to ES or clinical seizures in some cases (Figures 4, 10).

**Figure 9:**
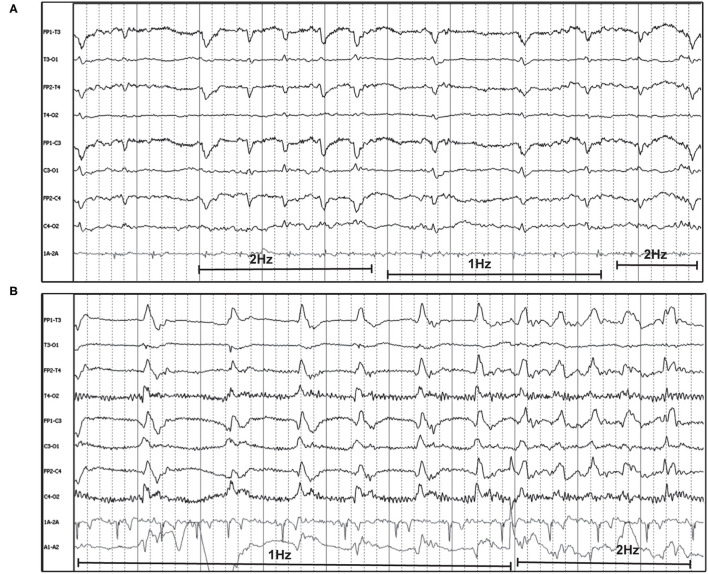
Two examples of fluctuating frequency of PDs in bipolar montage. **(A)** Ten year old domestic longhair cat with cluster seizures, unknown etiology (HF 70 Hz, sensitivity 10 uV/mm). **(B)** Two year old beagle with cluster seizures and MUO (HF 15 Hz, sensitivity 7 uV/mm). Only two changes in frequency are seen in this epoch, but frequency continued to fluctuate during the recording.

**Figure 10:**
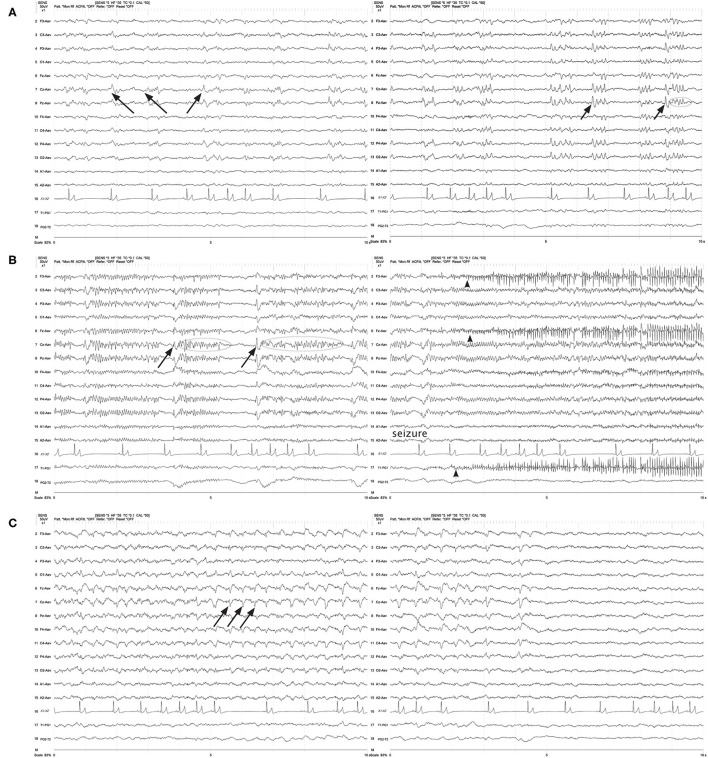
Three sets of consecutive EEG epochs in a 12 year old Shepherd mix illustrating evolution of GPDs to a focal seizure, and spontaneous resolution. The dog has a history of nasal aspergillosis and recent cluster seizures. Referential montage HF 35 Hz, sensitivity 5 uV/mm. **(A)** GPDs (arrows) progressively become more polyphasic, then develop bursts (>4 phases, longer than 500 ms) (circle). **(B)** GPDs (arrows) are associated with progressively longer bursts (circles), until a clinical focal seizure involving the left eye and face occurs, lasting 40 s. No pharmacologic intervention was given. Arrowheads indicate the start of electromyography (EMG) artifact from the muscle activity on the left frontal (F3, Fz) and left eye channels. **(C)** After termination of the clinical seizure, the GPDs (arrows) are apparent at a 2–2.5 Hz frequency, until they terminate spontaneously.

### 3.6. Response to intervention

If specific intervention during the EEG recording was done, it was typically an IV bolus of an antiseizure medication (ASM) (various drugs based on clinical decision, most commonly a benzodiazepine, phenobarbital, or levetiracetam). When IV ASMs were administered, many cases exhibited changes in the frequency and morphology of the PDs, but the discharges often persisted after drug administration, and in some cases returned to pre-ASM character with time if the medication had a short half-life (Figure 6).

## 4. Discussion

Even with hundreds of published cases of periodic discharges on EEG, with corresponding imaging, diagnosis, and outcome, there is still much debate in human medicine about the origin and clinical implications of various PDs, and particularly whether or not treatment with anti-seizure medication is indicated (9–11, 16, 17, 24, 34–36). In human medicine, PD frequency is known to change—either increasing or decreasing (8–11)—sliding up or down the IIC; PD waveform morphology can change—either spontaneously (11) or in response to intervention like surgery (37) or drugs (20, 21); and PDs are frequently documented to resolve with resolution or improvement of the underlying process (7, 8, 11, 19, 21). The case examples in this report demonstrated all of these characteristics. It is perhaps not surprising that dogs and cats can exhibit pathological periodic patterns comparable to those in people, since these species have been investigated for decades for similarities to humans in functional neuroanatomy (38–40).

Advances in invasive and non-invasive multimodal monitoring have not only increased detection of PDs in critically ill people, they have provided new information about the generators and pathophysiological processes associated with periodic discharges. Periodic discharges were originally thought to be secondary to white matter disease only, but human necropsy studies of patients with PDs on EEG showed cortical and/or subcortical gray matter lesions almost exclusively (41).

The origin of PDs is currently theorized as subsequent to interneuron exhaustion from repeated depolarization. The prolonged recovery of interneurons secondary to a structural lesion or frequent, intense depolarization (e.g., SE) results in disinhibition of adjacent local field potentials, permitting synchronization and generation of large-scale discharges, likely detected as PDs on scalp EEG (24, 42). Recent neural network modeling with disinhibition of synaptic connections as a parameter, reliably reproduces GPDs (43), and altering the patterns of disinhibition or the time delay in the model simulates GPDs with varying morphology (polyphasic, multiple waves). This is interesting pathophysiology to consider, given the variation in the morphology of the PDs both between different patients (Figures 1–3, 5, 6), and changes in PD morphology within the same patient, either spontaneously (Figures 4, 10) or with time and continued treatment (Figure 5). It is likely that the changes in PD morphology over time indicate re-establishment of more normal synaptic connections, and that the patient is “moving in the right direction,” down the IIC. In human EEG, the “+F” and “+R” superimposed additional rhythms (Figure 7) impart a higher likelihood of seizures, and the PDs with these additional features are more likely to be on the ictal side of the IIC (15). Therefore, simpler PDs likely indicate a more stable or less epileptogenic cortex, and are likely better for the patient. In case series of human patients with acute causes for PDs, over time, PD frequency decreased and the waveform morphology became less complex and longer in duration (wider waveform) before disappearing (8, 11). Similarly, in our cases where repeat EEGs were done, “simplification” or complete resolution of the PDs was noted (two cases illustrated in Figure 5) as the patient gradually improved clinically.

Visual examples of GPDs with triphasic morphology were specifically highlighted (Figure 8), since the origin, pathophysiology, and naming of this waveform morphology is one of the most debated of all the periodic patterns (44, 45). In human EEG, these previously named “triphasic waves” (TWs) were initially considered pathognomonic for hepatic encephalopathy (HE) (46). Over the years, however, many exceptions were noted, where TWs were present and the underlying process was not HE [toxins/medications (7, 20), immune-mediated encephalitis (47), among many others (44)], and there is no longer an assumption of a metabolic encephalopathy when TWs are noted. A human EEG study (48) evaluating inter-rater agreement on identification of GPDs and TWs found excellent inter-rater agreement for presence of GPDs, but only fair agreement for triphasic morphology. Interestingly, cases with TWs in this human study were less likely to have toxic/metabolic causes for their encephalopathy than those without TWs (48). Similar to the other adjustments in EEG terminology to avoid implying etiology, the current nomenclature describes these waveforms as “GPDs with triphasic morphology” (15), for which a metabolic encephalopathy would be one potential underlying cause (18, 44, 45). In the four cases of TWs illustrated in Figure 8, only one had HE (Figure 8D); the other cases had no systemic metabolic derangements, and seizures only. Conversely, the absence of GPDs with triphasic morphology does not exclude metabolic encephalopathy as an underlying disease: Figure 6 is an example of a dog with HE, seizures, and LPDs with a spike-wave morphology and not the prototypical triphasic morphology.

### 4.1. Frequency

Where a periodic discharge falls on the IIC strongly depends on its frequency. By the ACNS definition (15), any discharge ≥2.5 Hz is more likely to be an ictal pattern, whether a clinical seizure (patient has a synchronous physical manifestation) or solely an electrographic seizure (ES). A periodic discharge of ≤ 1 Hz is more likely interictal, and PDs with interdischarge intervals between 1 and 2.5 Hz have an increasing predisposition for seizures with the increasing frequency of the discharge (5, 6). In the two figures illustrating evolution of PDs, both the frequency and the complexity of the PDs increased, resulting in ES in one dog (Figure 4) and a clinical seizure in another (Figure 10).

### 4.2. Indicator or inciter of injury?

While the debate continues about whether PDs should be managed as ictal or interictal, as more is learned about the cellular pathophysiology of brain injury, a new question for consideration is whether PDs are solely sequelae to a cortical/subcortical injury, or are the depolarizations of the large volumes of neurons generating the PDs actually causing additional injury to the brain (5, 24). Some aspects of functional neuroimaging have contributed additional information (49). Magnetoencephalography (MEG) detects magnetic fields associated with current flow in neurons, and can facilitate further understanding of neural networks involved in generating PDs (50). MEG in patients with structural lesions and PDs shows LPDs originating from the interface region between the lesion and the normal brain—the “watershed” zone—not the lesioned brain itself, suggesting reactivity to the insult (50). Focal restricted diffusion on magnetic resonance imaging (rd-MRI) diffusion-weighted imaging (DWI) in patients with seizures results from increased metabolism and cytotoxic edema secondary to the ictal activity (51). A study of DWI and humans with LPDs on EEG with and without seizures, demonstrated rd-MRI only in the patients with seizure patterns and PDs, not in patients with PDs alone, suggesting that PDs alone do not cause restricted diffusion, the ictal activity does (52). Variable DWI changes are described in dogs with known history of clinical seizures (53), but timing of the imaging from the seizures (54) and the necessity of general anesthesia in veterinary patients for MRI likely alter the metabolic changes in the brain and thus the appearance on the imaging.

Witsch et al. (5) utilized invasive multimodal monitoring to show real-time changes in partial pressure of oxygen in interstitial brain tissue (PbtO2) in patients with PDs on EEG. They demonstrated a direct correlation with higher frequency PDs (>2 Hz) and tissue hypoxia, resembling the pathophysiologic changes seen with seizures. Very interestingly, their real-time monitoring of PbtO2 showed a decrease in brain tissue oxygenation that preceded increases in PD frequency, also suggesting that PDs on EEG are reactive to brain injury/hypoxia rather than causal (depending on the frequency). They acknowledge, however, that brain tissue hypoxia and PDs may also perpetuate a vicious cycle, initiated by hypoxia and exacerbated by higher-frequency PDs (5).

### 4.3. Outcome

Much of the historical literature on PDs in human medicine is in patients with documented seizures, other brain injury, or severe metabolic derangements (9, 18, 35, 38, 55, 56). Only recently have PDs been regularly identified in the critical care setting (1, 2, 6, 7). One case-controlled study (1) compared outcomes in two groups of patients: one with GPDs on EEG, and another group matched to the first set of patients based on disease etiology and level of consciousness, but without GPDs noted on EEG. GPDs were strongly associated with NCSE, and NCSE was associated with a poor outcome, however, the presence of GPDs alone was not specifically associated with a poorer outcome when compared to the case-controlled patients (1). Historically, in human medicine, PDs in patients with underlying pathology (i.e., not secondary to drugs/toxicity) are most commonly associated with a poor functional outcome or death (9, 35, 55–58).

The goal of this descriptive paper is to present several visual examples of periodic discharges in veterinary patients to increase recognition of these patterns for clinicians in neurology and critical care settings, and to encourage development of a common language for these periodic patterns to facilitate collaborative research. These visually distinctive waveforms are still hotly debated in human medicine, and the more that is known, the less black-and-white the understanding becomes: are PDs ictal or interictal? Should PDs be specifically treated? Does the presence of PDs predict a poor outcome? Are PDs a cause or consequence of brain injury? It seems like the answer to each of these questions is: “It depends…” Characteristics like higher frequency (>2 Hz), more complex waveforms (polyphasic, “+” modifiers), evolution of PDs, and presence of electrographic or clinical seizures are more supportive of an ictal pattern, while lower frequency (< 1 Hz), simpler PDs might be less likely to be associated with seizures, however, a patient's location on the IIC is not static. The presence of PDs on EEG seems to indicate a brain “on the edge:” whether on the edge of further decline or the edge of recovery is dependent on many factors.

This report illustrates that PDs occur in veterinary medicine and have the same features as those in human medicine. Next steps in veterinary medicine will be evaluating if the presence of PDs on EEG is correlated with clinical or electrographic seizure or status epilepticus, specific diagnoses, signalments, and outcomes. Optimally, we would learn which patterns and trends might benefit from treatment, and if that treatment improves patient outcome.

## Author contributions

DW, WB, MK, and KT contributed to the conception of the study. DW, WB, and MK selected EEG cases. KT, MK, and WB organized clinical information. MK wrote the first draft of the manuscript. All authors contributed to manuscript revision, read, and approved the submitted version.

## Supplementary material

Supplementary Figure 1 UC Davis electrode placement and montage.

Supplementary Figure 2 Bush Veterinary Neurology Service electrode placement and montage.
