# Supplementary material for: Periodic discharges in veterinary electroencephalography—A visual review
Source: Front Vet Sci. 2023 Jan 26;10:1037404. doi: 10.3389/fvets.2023.1037404 (PMC9909489; doi:10.3389/fvets.2023.1037404)
Supplement: Supplementary Figure 1 — UC Davis electrode placement and montage. [file Data_Sheet_1.PDF]

UCD montage

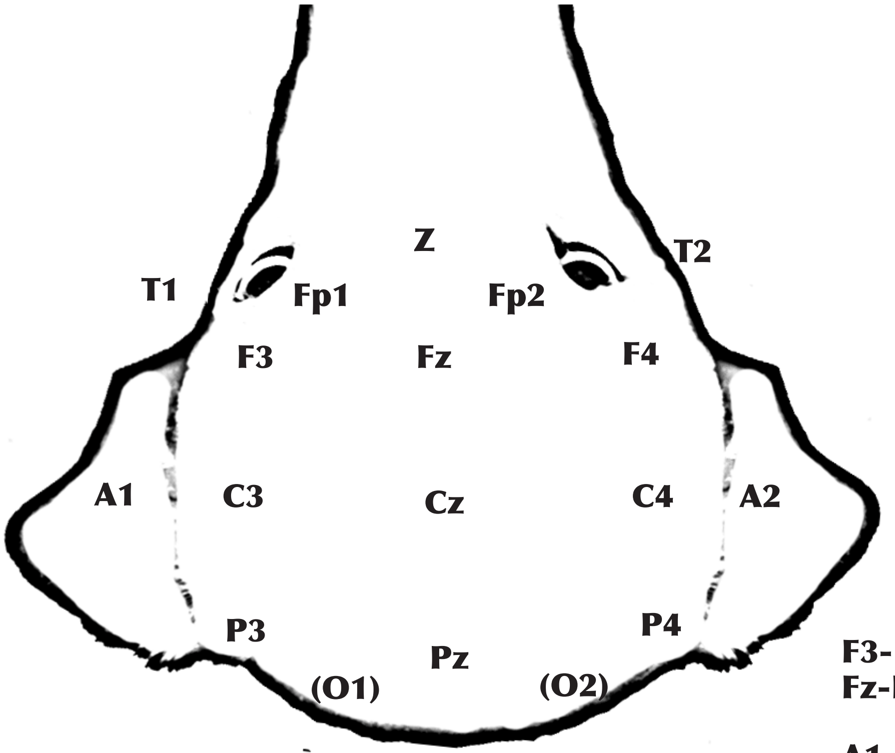

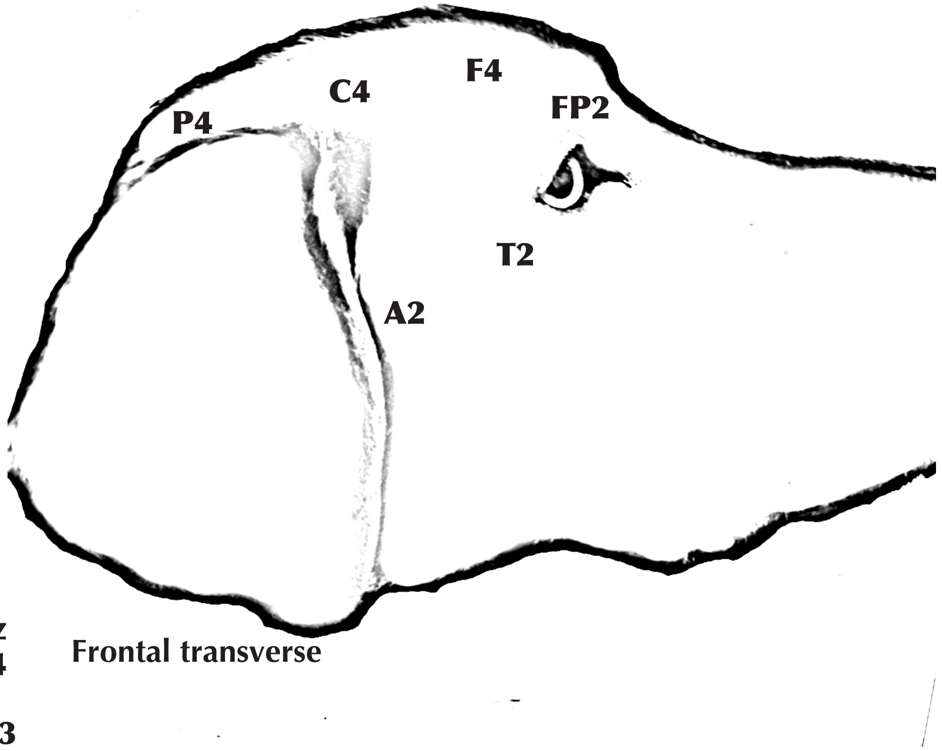

Z= ground

Placement of O1 & O2 was dependent on the size of the patient's head. Small breeds typically did not have these electrodes, and they were not included in the montage

F3-Fz<br>Fz-F4	Frontal transverse
A1-C3<br>C3-Cz<br>Cz-C4<br>C4-A2	Central/temporal transverse
P3-Pz<br>Pz-P4	Parietal transverse
F3-C3<br>C3-P3<br>(P3-O1)	Left longitudinal
Fz-Cz<br>Cz-Pz	Central longitudinal
F4-C4<br>C4-P4<br>(P4-O2)	Right longitudinal
Fp1-T1 - OS<br>Fp2-T2 - OD	Electro-oculograms
