# Supplementary material for: Periodic discharges in veterinary electroencephalography—A visual review
Source: Front Vet Sci. 2023 Jan 26;10:1037404. doi: 10.3389/fvets.2023.1037404 (PMC9909489; doi:10.3389/fvets.2023.1037404)
Supplement: Supplementary Figure 2 — Bush Veterinary Neurology Service electrode placement and montage. [file Data_Sheet_2.PDF]

BVS montage

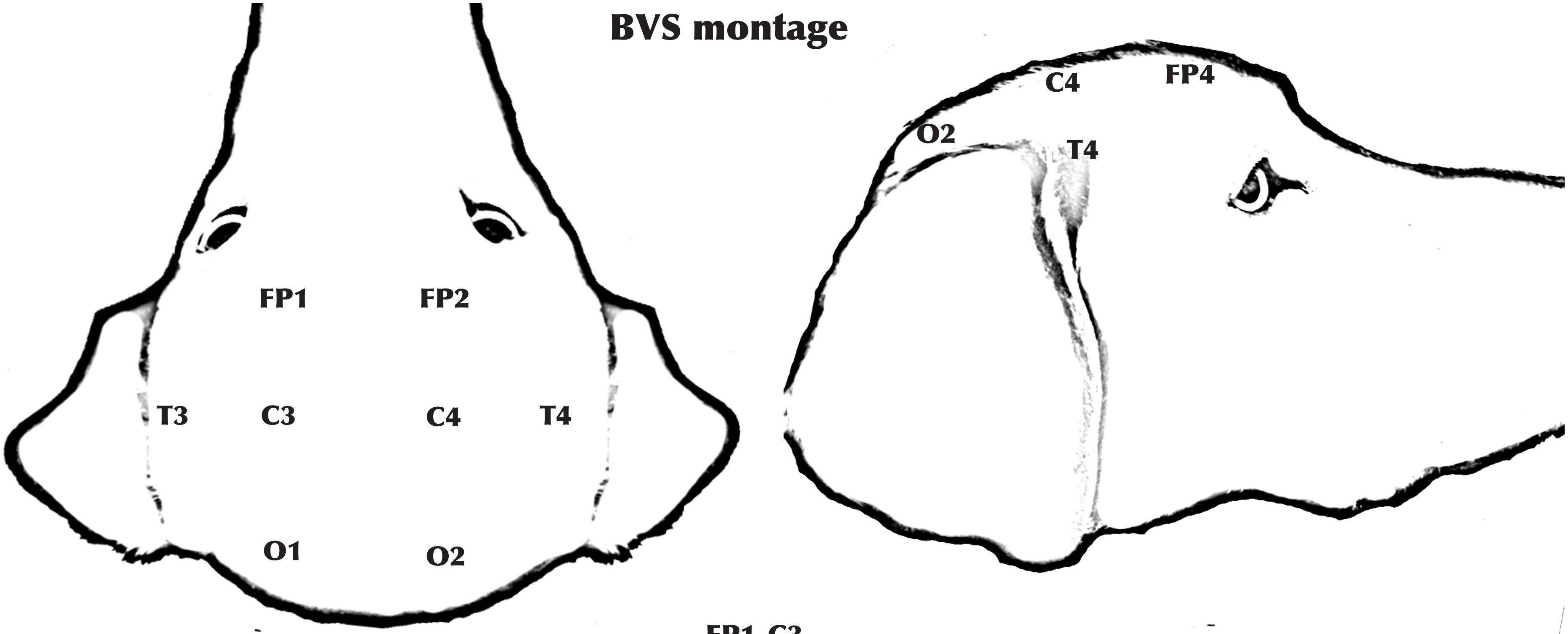

Z - placed over C2 vertebra

FP1-C3
C3-O1
Left parasagittal

FP2-C4
C4-O2
Right parasagittal

FP1-T3
T3-O1
Left temporal

FP2-T4
T4-O2
Right temporal

Z = ground
